# Classification of Sand-Binder Mixtures from the Foundry Industry Using Electrical Impedance Spectroscopy and Support Vector Machines

**DOI:** 10.3390/s24062013

**Published:** 2024-03-21

**Authors:** Luca Bifano, Xiaohu Ma, Gerhard Fischerauer

**Affiliations:** Chair of Measurement and Control Systems, Faculty of Engineering Science, University of Bayreuth, 95440 Bayreuth, Germany; luca.bifano@uni-bayreuth.de (L.B.); xiaohu.ma@uni-bayreuth.de (X.M.)

**Keywords:** electrical impedance spectroscopy (EIS), machine learning, support vector machines (SVM), feature analysis, classification, foundry, molding materials, sand

## Abstract

Molding sand mixtures used in the foundry industry consist of various sands (quartz sands, chromite sands, etc.) and additives such as bentonite. The optimum control of the processes involved in using the mixtures and in their regeneration after the casting requires an efficient in-line monitoring method that is not available today. We are investigating whether such a method can be based on electrical impedance spectroscopy (EIS). To establish a database, we have characterized various sand mixtures by EIS in the frequency range from 0.5 kHz to 1 MHz under laboratory conditions. Attempts at classifying the different molding sand mixtures by support vector machines (SVM) show encouraging results. Already high assignment accuracies (above 90%) could even be improved with suitable feature selection (sequential feature selection). At the same time, the standard uncertainty of the SVM results is low, i.e., data assigned to a class by the presented SVMs have a high probability of being assigned correctly. The application of EIS with subsequent evaluation by machine learning (machine-learning-enhanced EIS, MLEIS) in the field of bulk material monitoring in the foundry industry appears possible.

## 1. Introduction

The application of electrical impedance spectroscopy (EIS) as an analytical method is widespread. Examples include material characterization, monitoring and diagnosis of battery or accumulator systems, medical applications, or food monitoring [1]. The advantages of the measurement method include its non-invasive nature, the flexibility of the measurement duration and the volume under investigation, and the high information content due to the simultaneous determination of the real and imaginary parts of an impedance at various frequencies [1]. Impedance is an integral measure that contains information about larger volumes rather than local characteristics, which do not have to be statistically representative. Finally, the measurement is harmless to health compared to other methods such as X-rays [2]. For all these reasons, EIS is interesting for the in-situ monitoring of industrial processes. However, in order to be able to use EIS in an industrial environment, appropriate measuring devices are required, as laboratory equipment usually does not meet field requirements (it tends to be too expensive, too bulky, or too general-purpose). Various approaches to replacing laboratory equipment with application-specific developments can be found in the literature. These developments consist of a control unit, such as a field-programmable gate array [3], an Arduino DUE [4,5], or a Red Pitaya Board [6]. Conventional setups are extended and improved by, e.g., an impedance-to-digital converter [7], a logarithmic amplifier [4], or a digital auto-balancing bridge [8]. The operating frequencies range from 0.1 MHz [7] to 10 MHz [8], and the impedance magnitudes range from 100 Ω [8] to 10 GΩ [5]. The relative measurement deviations achievable with these application-specific developments are in the low single-digit percentage range [3,5,6,7,8]. Measurement times compatible with real-time requirements (between a few milliseconds and a few minutes) are achieved if the operating frequencies are restricted to sufficiently high values [8,9]. The devices developed in this way offer advantages since they can be adapted to the respective problem, such as battery technology [9], corrosion monitoring [5], and bioimpedance [3,6,8], and can also be inexpensive and compact compared to laboratory equipment [3,5]. Other research areas that are coming into the focus of EIS include environmental technology (microplastics detection [10], nitrate detection [11]), biotechnology or medical technology, especially sensor technology for minimally invasive surgical techniques, and cell monitoring [12,13,14]. In the medical field, the suitability of EIS for tomographic examinations is also being investigated [14,15].

The number of developments of suitable EIS devices for a wide range of applications demonstrates the interest and performance of EIS as a measurement technology in the field of online monitoring. An example of such an application is the process of used-sand regeneration in the foundry industry, in which used sand from casting production is processed so that the product obtained can be reused as a new-sand substitute for mold or core production [16] (pp. 311–313). The goal of regeneration is to reduce the raw material input of new quartz sand and the landfilling of used sand as a waste product. Such processes are currently controlled based on the empirical values of each foundry and with offline sand quality analysis. Process optimization in terms of energy consumption and product yield is highly desirable, but it is not at all clear how the continuous process state monitoring required for such a closed-loop system could be realized in the field and at a reasonable cost. We have investigated the merits of EIS as a measuring tool to identify the process moment at which regeneration is complete, and the processed former waste sand is available for reuse [17].

The EIS is intended to collect information on the used sand during the regeneration process. The composition of this sand varies depending on the casting process and the cast product. Typical main components can be quartz sand, chromite sand, and inorganic binder components such as bentonite [16] (pp. 19–65). Since these components are natural products, they vary in their properties, such as composition or particle size distribution. Some of the influence quantities affecting the measurement result have been characterized impedimetrically in the literature. For example, the influence of the water content of different types of bentonites on the dielectric properties was investigated in [18]. In addition to moisture, Szypłowska et al. [19,20] analyzed salinity and were able to extract a relationship between conductivity and permittivity via the salinity index model. The influence of the particle size distribution on the electrical impedance has also been analyzed in the literature. For example, Robinson et al. [21] mention its effect on the permittivity of the measured substance. In our previous EIS analyses of different sand types and mixtures, we were also able to show that the impedance spectra are characteristic of each mixture at the given particle size distribution of the mixtures [17,22,23].

The question that now arises is whether the characteristics or impedance features are suitable for distinguishing the two process states—“used sand to be regenerated” and “used sand regenerated sufficiently to serve as new-sand substitute”—from each other. This corresponds to a binary classification. An analytical solution to this task is difficult because of the large number of possible used-sand compositions and process parameters. For this reason, we have undertaken to solve the classification problem by machine learning (ML) in the form of support vector machines (SVM). An SVM has the potential to provide a generalized solution even though training data are available in limited quantities. Analytical problem-solving is no longer necessary. It outperforms numerical solution methods in computational speed [24]. The overfitting risk is low due to the robust mathematical design. The required computational power is also small due to efficient algorithms [25,26]. Extremely complex nonlinear input-output relationships can be handled with relative ease, and unique solutions can be found even with large data sets [26]. In addition, the risk of misclassification is minimized by maximizing the margin as outliers can be detected. The empirical training error is minimized [24,26].

The SVM methodology is the subject of ongoing research and development. The main focus is on efficiency improvements during training by avoiding numerical iteration procedures, a reduced memory requirement due to a smaller number of support vectors, and an optimized method for selecting the width of the kernel function by avoiding the grid search for determining the hyperparameters [27,28]. Approaches include the fast support vector classification or the semiproximal support vector machine [27,29]. Weakening the condition of a hard-margin to a soft-margin loss is also mentioned as a possible performance improvement [30]. Other approaches aim at improved algorithms that specifically reduce the input data to those that contain the most important pieces of information, leading to a higher training speed [31]. Typical SVM applications are in the field of image processing, i.e., surface, face, and object recognition or the analysis of handwriting and text classification [24,32]. Examples of more specific applications in medical technology include the analysis of ECG signals [33] and tumor detection [2,34]. More recent SVM applications are material classification tasks such as moisture determination in wood chips [35] or classification of various coals and rocks [36].

Our research follows on from material investigations, with the raw data being generated using EIS. Here, the input data for the SVM, denoted as features, are analyzed and selected both manually and automatically. The obtained results are then presented and compared within the context of this study. Importantly, employing effective feature selection techniques yields SVM models that demonstrate reduced complexity and a smaller risk of overfitting.

Based on the positive experiences described in the literature when investigating materials with EIS and the subsequent data analysis with SVM and based on the further investigation of the capabilities of SVM, we measured typical molding material mixtures from foundries impedimetrically and used the features extracted from the EIS output data for classification with SVM.

## 2. Materials and Methods

The molding materials investigated by us are listed in Table 1. They are typical materials for foundries. Quartz sand (MUT 1 and 2) or, in special applications, chromite sand (MUT 4) is used as the mold base material. To ensure that the mold has the necessary stability, various binder systems are used. Here, two inorganic systems based on bentonite were selected, which were added to the mold base material in different concentrations (MUT 5 and 6, both a–c). The two substances differed in terms of composition since the system of MUT 5 contained, in addition to the binder bentonite, further additives preset by the manufacturer. The binder system of MUT 6, on the other hand, was pure bentonite. MUT 3 is also quartz sand. Due to its lower quartz content, it does not meet the requirements of foundries, but it allowed us to test whether the proposed method can also distinguish quartz sands with varying quartz contents.

**Figure 1:**
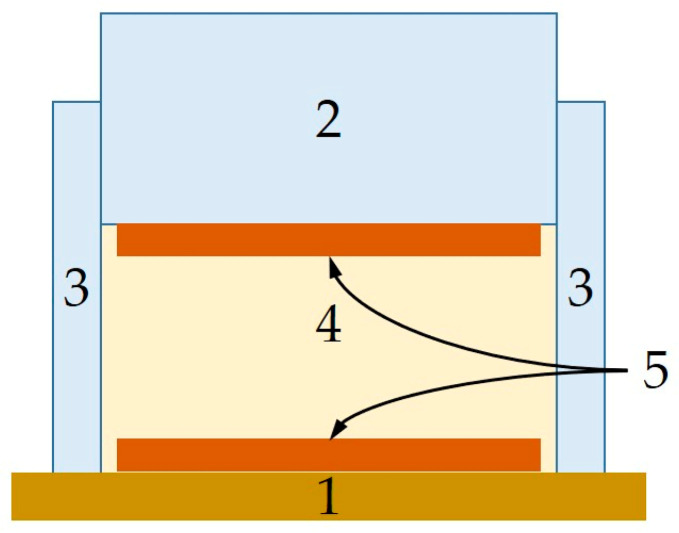
Cross-section of measurement cell used for sand characterization. (1) Wood base. (2) Macrolon stamp. (3) Polymer cylinder. (4) Material under test (MUT). (5) Copper electrodes (Area: 133 cm^2^. Distance: 4 cm) [22].

The molding compounds listed in Table 1 were each filled into a cylindrical plate-capacitor measuring cell (Figure 1), characterized, removed, filled in again, etc., and the whole cycle was repeated until ten fillings were characterized. The measurement of each individual filling involved a total of 20 repeated frequency sweeps so that at the end of the measurement series, a database of 10 × 20 frequency sweeps per MUT was available. The frequency range investigated included 145 frequency points between 0.5 kHz and 1 MHz. One sweep took approximately 1 min.

These settings were chosen with field applications in mind. The laboratory application of EIS usually involves lower frequencies, sometimes in the MHz range, and often frequencies higher than 1 MHz. Measuring low-frequency sinusoids, however, requires long measurement times, which is incompatible with the dynamics of an industrial process such as used-sand regeneration. In addition, higher-frequency signals are sensitive to disturbances in the field. In addition, they do not provide significant information in the current context.

Impedances were measured by an Agilent E4980A LCR meter. The measuring cell was connected to the instrument via two coaxial cables, and their shielding was connected to the ground LCR meter. Measurements were then performed at room temperature (between 21 °C and 26 °C). A broader temperature range is not necessary because the regeneration in the foundry is performed at temperatures between 20 °C and 40 °C. Impedimetric investigations of the MUTs in this temperature window did not reveal any significant influence of the temperature on the data.

## 3. Results and Discussion

### 3.1. Overview of the Raw Impedance Data

**Figure 2:**
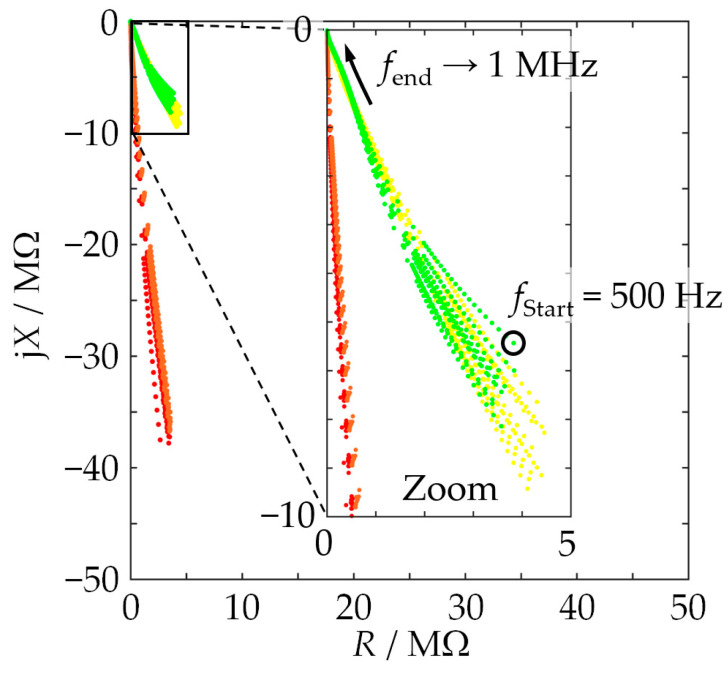
Impedance–locus plot of ten impedance spectra (raw measurement data): MUT 1 (red), MUT 2 (orange), MUT 3 (yellow), MUT 4 (green).

To illustrate the characteristics of the measured impedances, Figure 2 shows ten spectra randomly selected from all spectra obtained with pure substances, MUTs 1 through 4. The following aspects can be identified:

- The details of the impedance locus are characteristic for each cell filling, i.e., for each MUT.
- The more similar the MUTs are in their composition, the more difficult it is to differentiate the impedance curves (cf. MUTs 1 and 2).
- The dependence of the complex permittivity on the grain size distribution mentioned in the introduction shows up in a similar dependence on the impedance. For example, the impedance–locus curves for MUTs 1 and 2 are slightly shifted against each other.
- It does not suffice to merely look at the shape of an impedance locus. The frequency associated with each point of the locus is also important. Otherwise, by way of an example, one could hardly distinguish MUTs 3 and 4 as their impedance–locus curves without frequency labels along the curves are quite similar.
- The largest relative standard uncertainty occurring in Figure 2 appears at 500 Hz with a value of 7.9% for the resistance and 1.8% for the reactance. The EIS data are, therefore, deemed suitable for evaluation by machine learning.

### 3.2. Feature Generation

It is conceivable, of course, to use the raw EIS data as direct input to an ML algorithm, e.g., a classifier [2]. For large data sets, this can turn out to be computationally expensive both during the algorithm training and during its field use. The approach to completely turn over the data interpretation to an algorithm also involves risks. One cannot be sure what exactly an ML algorithm learns from training data, and it is difficult to gain any physical insight from its results. We, therefore, took a different approach. Instead of passing raw EIS data to an SVM classifier, we extract m features from the n raw data (with n>m) to be passed to the classifier. This leads to SVMs that are computationally less expensive, especially with large data sets, and faster to train. It is not quite what is called physics-informed ML but may be considered to be data-quality restricted ML.

**Figure 3:**
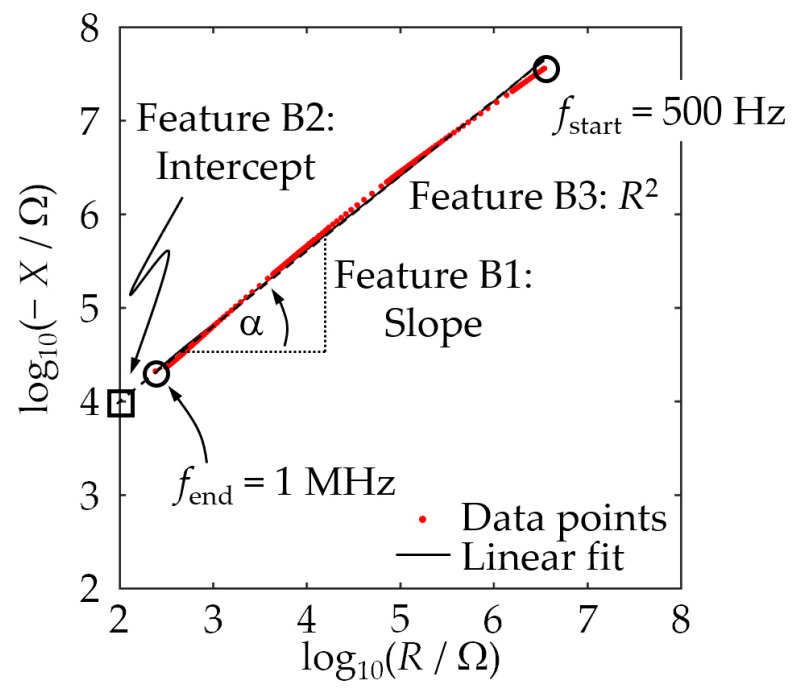
Impedance–locus plot, in double-logarithmic representation, for a measurement cell filled with MUT 1 (circles) and linear regression line (solid curve). The three extracted class-B features—“slope”, “intercept”, and “coefficient of determination”—are also visualized.

We focused on two types of features: statistical features of impedance spectra on the one hand (feature class A, [37]) and parameters of approximation curves fitted to the impedance–locus curves on the second hand (feature class B, [37]). The four features belonging to class A used in our studies were the median and the mean of both the measured resistance and the measured reactance of a MUT sample over the entire frequency range (see [17]). The three features belonging to class B were the slope, the intercept with the vertical axis, and the coefficient of determination of a regression line for the measured impedance–locus curve of a MUT sample in double-logarithmic representation. Figure 3 visualizes the result of fitting a line to an impedance curve section in double-logarithmic representation to convey an idea of why such a linear fit produces meaningful features in the given context.

### 3.3. Classification with SVM

We used the Classification Learner as part of the Statistics and Machine Learning Toolbox 12.1 of MATLAB R2021a [38] to generate SVMs, which were adapted to classify MUTs of the type considered in this work. A total of 70% of the available data were used for training and 30% for testing. To check the robustness of the SVM with respect to possible outliers in the measurement data, the data were randomly divided into training and test data ten times. The following list provides an overview of this grouping:

- 10 MUTs investigated.
- Per MUT: 200 measurement repetitions (of which 140 were used for training and 60 were used as test data).
- Per impedance spectrum: 7 features (four of class A and three of class B).
- Together, this yielded 10 × 200 × 7 = 14,000 feature values.
- In an experiment based on $n_{f}$ out of the 7 features ($n_{f}=1\ldots 7$), the corresponding subset of 10 × 200 × $n_{f}$ = 2000 × $n_{f}$ feature values was then randomly split into 0.7 × 2000 × $n_{f}$ = 1400 × $n_{f}$ feature values for training and 0.3 × 2000 × $n_{f}$ = 600 × $n_{f}$ feature values for testing. Such splits were made 10 times per experiment.

The parameter settings used to generate the ten SVMs are listed in Table 2.

For each feature described in Section 3.1, SVMs were generated with the parameters listed in Table 2 and in the manner described in the bullet list above ($n_{f}=1$). In the following, for brevity’s sake, we only discuss the results for the feature “median resistance” in detail. Only the final results are presented for all other features.

The confusion matrix obtained for the feature “median resistance” with the selected data is shown in Table 3. The confusion matrix columns list the MUTs predicted by the SVM, whereas the rows list the MUTs actually present. The entries on the diagonal are the numbers of correct classifications, and off-diagonal entries are the numbers of wrong classifications. For example, in 140 out of 140 cases, MUT 1 was correctly identified by the SVM. MUT 2 was correctly identified in 110 out of 140 cases but was mistaken for MUT 5a in 20 cases, for MUT 6a in 2 cases, and for MUT 6c in 14 cases. The shaded rows in Table 3 indicate those MUTs that the SVM could not identify correctly in all cases. The misclassifications involve very similar mixtures of substances. Such cases may pose problems for classifiers based on a single feature only. Their classification quality is still quite good but not as excellent as when the MUTs differ significantly. The assignment correctness of this training data set results in 90.9%.

Each of the available data sets yields a slightly different confusion matrix and slightly different assignment correctness since the assignment of the data to the training or test data varies slightly. It follows that the result of the SVM, i.e., the assignment correctness and thus the probability of misclassification, depends on the choice of data. To be able to describe the quality of the SVM better and independently of the data sets, we, therefore, used the ten confusion matrices with the corresponding assignment correctnesses and performed an uncertainty analysis by calculating the standard uncertainty $u=s/\sqrt{10}$ with s the standard deviation according to [39] (p. 10). This then led to a complete measurement result for the correctly assigned data of the confusion matrix, i.e., the diagonal elements, and for the assignment correctness. The determined ten assignment correctnesses of the ten data sets for the feature “median resistance” are listed in Table 4.

From the assignment correctnesses of Table 4, a mean assignment correctness of 91.2% and an absolute standard uncertainty of 0.14% are then obtained for the ten training data sets evaluated. Therefore, the complete measurement result for the assignment correctness () with the training data when using the feature “median resistance” is ${AC|}_{Median\text{-}R}=91.2\%\pm 0.14\%$. Following the same procedure, the corresponding values for the other features and test data were obtained, as shown in Table 5.

Using a confusion matrix of the averaged values of the assignment correctness and a matrix of the standard uncertainty elements matching the confusion matrix, it is then possible to estimate more precisely how often a particular misclassification may occur. These two matrices for the training data and the feature “median resistance” are shown in Table 6 and Table 7.

The comparison of Table 6 and Table 7 reveals that most of the training data are assigned to the correct MUT class using the feature “median resistance”. The results are worse for MUT 6c. This concerns both the mean value of all data correctly assigned to this class (100 is the lowest value among all diagonal elements in Table 6) and the standard uncertainty (1.28% is the largest value of all correctly assigned data, i.e., all diagonal elements, in Table 7). Thus, improving the classification quality via a combination of different features to generate an SVM must be the goal.

### 3.4. Combined Features for Classification

The classification of similar mixtures of substances, which proved difficult when based on a single feature only, should be more successful when two or more features are passed to a classifier $n_{f}\geq 2$. We investigated the benefits of combining two features $n_{f}=2$ when training SVMs with the settings from Table 2. The resulting mean values of the assignment correctness and the standard uncertainty are listed in Table 8. As in the previous section, the assignment correctnesses within ten different data sets were determined for the three feature combinations shown in Table 8 and then averaged for each feature combination. Again, the standard uncertainty as a measure for the variance of each assignment’s correctness was determined for each investigated feature combination. The training confusion matrix for the feature combination “median resistance + median reactance” for the mean values is shown in Table 9.

**Figure 4:**
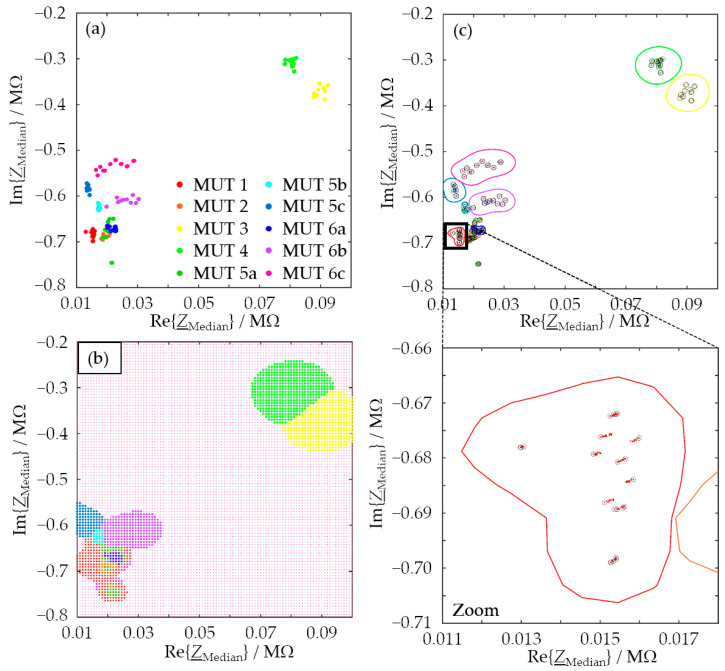
SVM classifier operating in the 2D feature space spanned by the median resistance and the median reactance for one training data set. (**a**) Distribution of the data points. (**b**) Division of the feature space into regions associated with the ten different MUT classes. The largest region is taken up by MUT 6c (color: magenta). (**c**) Data points with associated decision boundaries. The points marked by black circles are the support vectors defining the decision boundaries. The zoomed-in region contains data points belonging to MUT 1.

The assignment correctness assumed a value of 99.7% in the worst case, i.e., the data could be assigned to the individual MUT classes very reliably via the feature combinations presented. Overall, it can be stated that the use of two features improved the classification quality significantly. In the case of the feature combination used for Table 9, the SVM only had misclassifications for MUT 2 and MUT 5a (rows shaded in gray). The occasional confusion of MUT 2 with MUT 5a or 6 and vice versa results from the similarity of these mixtures in terms of chemical composition. It may well be that the recorded impedances sometimes are too similar to safely allow discrimination. The results for one training data set shown in Figure 3 and Figure 4 support this assumption.

The distribution of measurement points in the feature space spanned by the median resistance and the median reactance, Figure 4a, already suggests that most of the points belonging to a given MUT are separable from the rest. However, the feature ranges of MUTs 2, 5a, and 6a overlap, which may have led to the misclassifications documented in Table 9. Figure 4b graphically shows how the SVM generated with the training data divides the feature plane into ten distinct domains and assigns each domain to one MUT. The color code of the respective domain is based on the colors assigned to the individual MUTs in Figure 4a. Programmatically, the domain-to-MUT mapping was solved in such a way that a binary SVM was generated for each MUT, which had to distinguish between two material classes, the selected MUT and all the rest (one vs. all). In a loop, the procedure was applied to all MUT classes, and a total of ten binary SVMs were generated, with each individual SVM providing the corresponding decision boundary of the associated MUT. The decision boundaries are shown in Figure 4c by colored lines matching the colors of the associated MUTs. The feature points marked by black circles are the so-called support vectors that define the decision boundaries. The number of support vectors is related to the choice of the box constraint: The smaller the box constraint is chosen, the more points are support vectors, i.e., the higher the probability of overfitting. Such overfitting is detrimental because the generated SVM then classifies the training data particularly well but produces less than optimum results for any other data not part of the training. In this application, the box constraint was chosen as 1, and this avoided overfitting. This can be seen from the smooth shape of the decision boundaries and is also confirmed by the values of the assignment correctness of the test results (Table 5 and Table 8). The latter are in the same range as the corresponding values for the training data, although the SVM has never seen the test data before the actual test.

**Figure 5:**
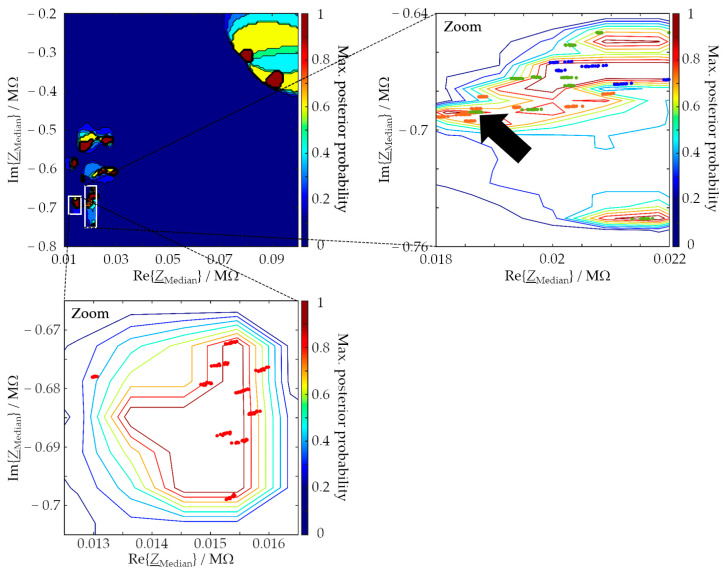
Probability ranges predicted with the generated SVM operating in the feature space spanned by the median resistance and the median reactance of one data set (same EIS input data as for Figure 4). The zoomed-in region at the bottom contains the data points produced with MUT 1. The zoomed-in region on the right shows the prediction probabilities in the area of the data of MUT 2 (orange), MUT 5a (green), and MUT 6a (blue). The black arrow marks those points of MUT 5a, which are located in the area of class MUT 2 and therefore are misclassified.

It is noticeable that the areas enclosed by the decision boundaries in Figure 4c are smaller than the color-coded assignment domains in Figure 4b. The reason for this is that all data points in the area enclosed by the decision boundary are assigned to the associated MUT class in any case. Points that lie outside this range can be assigned to different MUT classes. Which class they are then assigned to depends on the probability of assignment to a class that the generated SVM calculates for these points, i.e., the posterior probability. Figure 5 represents this assignment probability for the chosen feature space. This representation allows statements about the probability that an arbitrarily chosen point in the feature space belongs to one of the MUT classes. Large parts of the region marked in magenta in Figure 4b (MUT 6c) have a low assignment probability of less than 20%. It follows for data points that fall into this region of the feature space that they are assigned to class MUT 6c, but there is only a low probability that they actually belong to class MUT 6c.

Zooming into the region of MUT 1 shows that, as expected, data points associated with unknown MUTs are more likely to be classified as MUT 1 the closer they are to the MUT-1 training data. As a result, the decision boundary of the MUT 1 class is similar to this in Figure 4c. The zoomed-in probability range of the feature data of MUTs 2, 5a, and 6a shows why it is difficult to classify the three MUTs unambiguously based on the two selected features. The data lead to very similar feature values due to the very similar chemical composition of the three MUTs (see Table 1). As a consequence, and by way of an example, the data points of MUT 5a marked with an arrow in Figure 5 are misclassified as MUT 2. More than two features are required to prevent such misclassification.

### 3.5. Results with Automated Feature Selection

The features to be used for an SVM can be selected by known algorithms, e.g., Sequential Feature Selection (SFS) [40]. SFS is a method employed to select the most valuable features based on a criterion, such as the minimum classification error of the SVM constructed from the training data. The procedure starts with the creation of a random nonstratified partition for a 10-fold cross-validation on all observations, amounting to the 2000 impedance spectra in our specific case.

The algorithm sequentially builds a candidate set by adding features one at a time. The feature that exhibits the best performance, as indicated by the smallest cross-validated criterion, becomes the initial member of the candidate set. This process iterates for each remaining feature, generating new candidate sets by adding the corresponding feature to the existing candidate set. The cross-validated criterion for each new candidate set is then computed and removed. The feature that causes the most significant improvement of the model performance, notably by reducing the cross-validated criterion value, is added to the candidate set.

This iterative procedure continues until adding a feature no longer results in a substantial improvement (i.e., decrease) in the criterion value beyond the termination tolerance, set at 10^−6^ in our case.

According to the SFS results, the first and most valuable feature was the intercept of the regression line, with a cross-validated criterion value of 0.00104. This agrees with Table 5, in which the highest assignment correctness is documented for the intercept of the regression line as a feature. The combination of intercept with median reactance led to the highest assignment correctness among all two-feature combinations (cross-validated criterion value below 10^−6^). These two features alone could perfectly classify all observations. The addition of further features did not contribute significantly to the classification task.

To check on this result, the original dataset was once more randomly divided into training and test data at a ratio of 70% to 30%. For the two features selected through SFS, an SVM was generated with the parameters listed in Table 2. The test results demonstrate a remarkable 100% classification accuracy.

## 4. Conclusions

This contribution is concerned with the real-time assessment of molding-compound quality in field environments of the foundry industry. The task involves (a) the generation of measurement data, (b) the interpretation of the measurement data, and (c) some measure for the overall soundness of the interpretation. Task (a) was solved by EIS. This then means that entire impedance spectra (or, put differently, complex-valued frequency series) form the basis of step (b).

Step (b) was tackled by machine learning in general and by SVM in particular. In principle, SVM can be expected to be suitable for sand classification based on EIS spectra, but we have actually demonstrated it and have now investigated the key points that need to be considered. Feature candidates to be used for classification by the SVM were the median resistance, the mean resistance, the median reactance, and the mean reactance of an entire impedance spectrum (feature class A), as well as the fit parameters slope, intercept, and coefficient of determination of a linear fit of the logarithmized measurement data (feature class B). Convincing assignment results (>83%) were achieved even with single features, with the median resistance performing particularly well (>90%). Misclassifications could be explained by similar compositions of two or more molding materials.

To achieve even better assignment results, two features were combined, and a new SVM was generated for each case. As a result, the overall assignment correctness could be increased for all feature combinations examined so that values of over 99% were achieved. As we can show, by combining the features with the highest information density determined by an SFS, viz., the intercept of the regression line and the median reactance, one can achieve an assignment correctness of 100%.

Regarding task (c) from above, variance analysis of the SVM results has shown that the uncertainty can be a valuable quality criterion for an SVM result in addition to the assignment correctness. The assignment correctness can be high overall, although the uncertainty of any one class assignment is also high. It is our understanding that measurement results involving some form of machine learning cannot be trusted (in the sense that led to the GUM methodology) unless a variance analysis or an equivalent approach is performed. At least for the material systems studied by us to date, we believe that we can state the following: EIS spectra are suitable input data to SVM classifiers, which are able to classify molding compounds reliably and with a small enough uncertainty to be useful for foundry applications.

In further steps, the SVM will be optimized, i.e., an optimal hyperparameter selection will be performed. With the results generated in this process and the classification findings, the aim will then be to refine the classes in order to be able to make concentration statements about how much bentonite is present in the measured mold material via a regression SVM. This aspect is highly relevant in the field of molding material preparation in foundry applications.

## Figures and Tables

**Table 1:** Chemical composition of the molding materials was investigated. MUT 1 and 2 are quartz sands with 0.2 mm and 0.4 mm mean grain size, respectively (manufacturer’s data). MUT 3 is also quartz sand but with lower quartz content and a mean grain size of 0.3 mm. MUT 4 is a chromite sand with a mean grain size of 0.3 mm. MUT 5 and 6 are mixtures of MUT 1 and different prefabricated typical binder mixtures. MUT 1 and MUT 6 have been used in previous investigations [17], and MUT 4 in [23]. Bentonite is denoted as “B”, and other additives are denoted as “A”.

MUT	Mass Fraction in %							
	SiO_2_	Al_2_O_3_	Cr_2_O_3_	Fe_2_O_3_	MgO	K_2_O	B	A
1	99.53	0.01	0	0.01	0.01	<0.01	0	0
2	99.53	0.01	0	0.01	0.01	<0.01	0	0
3	84.6	7.63	0	0.21	0.34	5.35	0	0
4	0.7	14.8	46.4	28.2	9.5	0	0	0
5a	98.53	<0.01	0	<0.01	<0.01	<0.01	0.6	0.4
5b	94.55	<0.01	0	<0.01	<0.01	<0.01	3	2
5c	89.58	<0.01	0	<0.01	<0.01	<0.01	6	4
6a	98.53	<0.01	0	<0.01	<0.01	<0.01	1	0
6b	94.55	<0.01	0	<0.01	<0.01	<0.01	5	0
6c	89.58	<0.01	0	<0.01	<0.01	<0.01	10	0

**Table 2:** Parameters used to generate SVMs.

Parameter	Setting
Model type	Fine Gaussian SVM
Cross-validation	10-fold
Kernel function	Gaussian
Box constraint level	1
Kernel scale mode	Auto
Multiclass method	One-vs-One
Standardize data	True
Loss function	Hinge loss

**Table 3:** Confusion matrix of the training data for the feature “median resistance” for one chosen distribution.

True MUT	Predicted MUT									
	1	2	3	4	5a	5b	5c	6a	6b	6c
1	140	0	0	0	0	0	0	0	0	0
2	0	110	0	0	21	0	0	5	0	4
3	0	0	140	0	0	0	0	0	0	0
4	0	0	0	140	0	0	0	0	0	0
5a	0	20	0	0	110	0	0	9	0	1
5b	0	0	0	0	0	138	0	0	0	2
5c	0	0	0	0	0	0	140	0	0	0
6a	0	2	0	0	11	0	0	123	0	4
6b	0	0	0	0	8	0	0	1	129	2
6c	0	14	0	0	1	0	0	16	6	103

**Table 4:** Assignment correctness AC in % for the application of the feature “median resistance” to ten different training data sets.

Data Set No. *i*	1	2	3	4	5	6	7	8	9	10
AC*_i_*/%	90.9	91.1	90.6	90.7	91.6	91.6	91.9	91.2	91.6	90.9

**Table 5:** Means and standard uncertainties of assignment correctness for all features and broken down by training and test data.

Feature Class	Feature	Training Data		Test Data	
		Mean in %	Std. unc. in %	Mean in %	Std. unc. in %
A	Median resistance	91.2	0.14	90.6	0.28
A	Median reactance	88.9	0.19	88.5	0.46
A	Mean resistance	86.6	0.24	87.0	0.54
A	Mean reactance	84.8	0.23	85.3	0.60
B	Slope of regression line	83.1	0.23	82.8	0.56
B	Intercept of regression line	92.3	0.29	92.0	0.33
B	Coefficient of determination of regression line	88.1	0.29	87.3	0.52

**Table 6:** Confusion matrix of the mean values of all training data sets for the feature “median resistance”.

True MUT	Predicted MUT									
	1	2	3	4	5a	5b	5c	6a	6b	6c
1	140	0	0	0	0	0	0	0	0	0
2	0	111	0	0	19	0	0	3	0	8
3	0	0	140	0	0	0	0	0	0	0
4	0	0	0	140	0	0	0	0	0	0
5a	0	19	0	0	114	0	0	6	1	1
5b	0	0	0	0	0	139	0	0	0	1
5c	0	0	0	0	0	0	140	0	0	0
6a	0	1	0	0	12	0	0	123	1	4
6b	0	0	0	0	5	0	0	1	131	4
6c	0	14	0	0	1	0	0	16	9	100

**Table 7:** Matrix of the absolute standard uncertainty elements in % of the confusion matrix of means from Table 6.

True MUT	Predicted MUT									
	1	2	3	4	5a	5b	5c	6a	6b	6c
1	0	0	0	0	0	0	0	0	0	0
2	0	0.99	0	0	0.82	0	0	0.62	0	0.50
3	0	0	0	0	0	0	0	0	0	0
4	0	0	0	0	0	0	0	0	0	0
5a	0	0.81	0	0	0.99	0	0	0.72	0.26	0.15
5b	0	0	0	0	0	0.21	0	0	0	0.21
5c	0	0	0	0	0	0	0	0	0	0
6a	0	0.21	0	0	0.74	0	0	0.81	0.17	0.26
6b	0	0	0	0	0.69	0	0	0.20	0.86	0.88
6c	0	0.78	0	0	0.16	0	0	0.60	0.67	1.28

**Table 8:** Assignment correctness when using a combination of two features to classify MUTs.

Feature Combination	Training Data		Test Data	
	Mean in %	Std. unc. in %	Mean in %	Std. unc. in %
Median resistance + median reactance	99.7	0.04	99.7	0.07
Mean resistance + mean reactance	100	0.00	100	0.00
Slope + intercept of regression line	100	0.00	100	0.00

**Table 9:** Confusion matrix of the training data for the feature combination “real and imaginary part of the median” applied to the mean values of all data sets.

True MUT	Predicted MUT									
	1	2	3	4	5a	5b	5c	6a	6b	6c
1	140	0	0	0	0	0	0	0	0	0
2	0	139	0	0	1	0	0	3	0	0
3	0	0	140	0	0	0	0	0	0	0
4	0	0	0	140	0	0	0	0	0	0
5a	0	4	0	0	136	0	0	0	0	0
5b	0	0	0	0	0	140	0	0	0	0
5c	0	0	0	0	0	0	140	0	0	0
6a	0	0	0	0	0	0	0	140	0	0
6b	0	0	0	0	0	0	0	0	140	0
6c	0	0	0	0	0	0	0	0	0	140

## Data Availability

The data are accessible online with the following DOI: https://doi.org/10.15495/do_ubt_2059.
